# Pyogenic Hepatic Abscess as a Complication of Appendicitis: A Case Report

**DOI:** 10.1002/ccr3.72037

**Published:** 2026-02-24

**Authors:** Mohammad AshrafAzimi, Abbas Abdollahi, Mohammad karimi, Homa Jajarmi, Ali Mehri

**Affiliations:** ^1^ Department of General Surgery, Faculty of Medicine North Khorasan University of Medical Sciences Bojnord Iran; ^2^ Endoscopic and Minimally Invasive Surgery Research Center, Ghaem Hospital Mashhad University of Medical Sciences Mashhad Iran; ^3^ Faculty of Medicine North Khorasan University of Medical Sciences Bojnurd Iran; ^4^ Department of Pediatrics, Faculty of Medicine North Khorasan University of Medical Sciences Bojnord Iran

**Keywords:** acute appendicitis, percutaneous drainage, pyogenic liver abscess, septic shock

## Abstract

Pyogenic liver abscess is a rare and under‐recognized complication of gangrenous or perforated appendicitis in children. Management typically requires a combination of antibiotic therapy and abscess drainage. Although uncommon in the pediatric population, pyogenic liver abscess is more frequently observed in the elderly people. With an aging population, it is increasingly important for young physicians to recognize its clinical presentation and treatment strategies. This report describes an 11‐year‐old boy admitted with fever, impaired consciousness, nausea, and vomiting—initially raising concern for meningitis. A CT scan revealed a hepatic abscess and findings consistent with acute appendicitis. Following multidisciplinary consultation, the patient underwent percutaneous drainage of the liver abscess and an appendectomy. His postoperative course was uneventful, and he was discharged with a liver drain in place. Given the limited reliability of history and physical examination in pediatric patients with septic shock—especially in emergency settings—clinicians must maintain a broad differential diagnosis.

## Key Clinical Message

In children with septic shock or atypical presentations, pyogenic liver abscess should be considered as a rare complication of appendicitis. Early imaging and prompt multidisciplinary management, including abscess drainage and targeted antibiotic therapy, are essential for accurate diagnosis, effective treatment, and favorable clinical outcomes.

## Graphical abstract

Pyogenic liver abscess management.

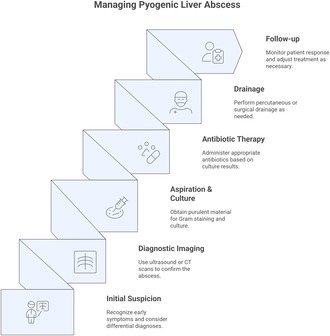

## 1 Introduction

Acute appendicitis is a common intra‐abdominal condition, with a reported mortality rate of 6%–9%, although its exact etiology and epidemiology remain unclear. Proposed contributing factors include mechanical obstruction, low dietary fiber intake, familial predisposition, socioeconomic status, poor quality of life, and bacterial, viral, or parasitic infections. Appendicitis is also a frequent indication for abdominal and pelvic computed tomography (CT) imaging. The rate of appendiceal perforation is estimated at 10%–20% [1].

Pyogenic liver abscesses are typically classified according to their presumed route of hepatic invasion, including the biliary tree, portal vein, hepatic artery, direct extension from adjacent infections, or penetrating trauma [2]. Most appendiceal perforations occur intraperitoneally due to the accumulation of inflammatory fluid or mucus around the inflamed appendix. One important, although nonspecific, complication of acute appendicitis is the development of single or multiple pyogenic liver abscesses [3].

The venoportal system drains nearly all abdominal viscera. Phlebitis, defined as purulent thrombosis of the hepatic veins or their branches, secondary to conditions such as diverticulitis, pancreatitis, encephalitis, inflammatory bowel disease, or postoperative infections, can result in liver abscess formation. Although the incidence of pyogenic liver abscess has declined due to widespread antibiotic use, untreated appendicitis remains a notable cause [4].

Another common mechanism involves the spread of organisms into the bile ducts, as observed in cholecystitis or ascending cholangitis. A third route is the direct implantation of pathogens into hepatic tissue, typically through iatrogenic means such as biopsy or following penetrating trauma [5].

Additional causes include cholangitis, subphrenic and perinephric abscesses, and various other intra‐abdominal infections. Penetrating liver trauma can lead directly to abscess formation, whereas nonpenetrating injuries may result in hematomas that predispose to secondary bacterial infection. Predisposing factors also include hepatic damage from tuberculosis, tumor necrosis, or cirrhosis. Host‐related factors are particularly important in cryptogenic cases. Systemic conditions such as diabetes, cardiopulmonary disease, malignancy, and cirrhosis are common comorbidities. Notably, diabetes increases the risk of pyogenic liver abscess by up to threefold, and alcohol use is also associated with increased susceptibility [6].

Neutrophil‐related disorders, including chronic granulomatous disease (CGD) and Job's syndrome, are associated with liver and systemic abscess formation. Patients with hemochromatosis have a heightened risk, particularly of *Yersinia enterocolitica* infection leading to hepatic abscess [7]. We present an unusual case of a persistent pyogenic liver abscess secondary to subacute or unrecognized appendicitis.

## 2 Case History/Examination

An 11‐year‐old male patient presented with fever, altered consciousness, nausea, and vomiting, initially raising concern for meningitis. A cerebrospinal fluid (CSF) analysis was performed, and empirical broad‐spectrum antibiotics were initiated.

**FIGURE 1:**
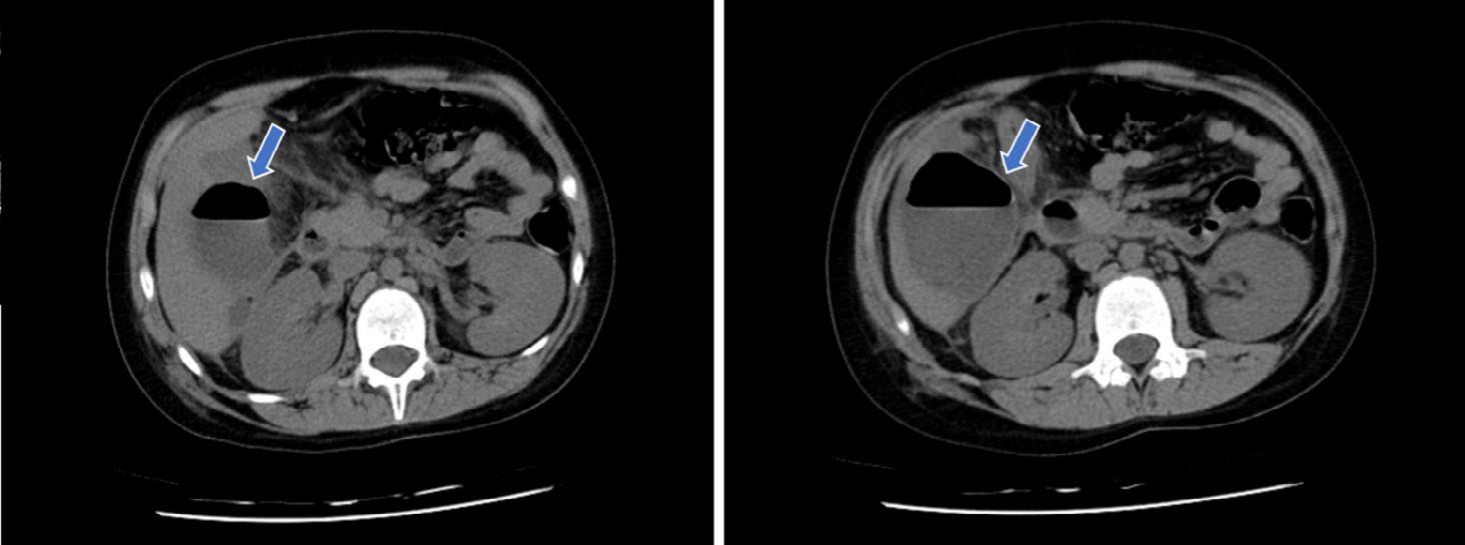
Axial abdominal CT scan images of the patient. The arrows indicate the hepatic abscess.

Due to the absence of clinical improvement and the patient's critical condition, and because initial clinical findings did not support targeted ultrasonographic evaluation, a non‐contrast abdominal and pelvic CT scan was performed on hospital day six. Imaging revealed a subcapsular abscess in segment VI of the liver, measuring 49 × 52 × 61 mm, with a visible fluid–air level. The abscess extended toward the ascending colon, resulting in obliteration of the intervening fat plane. The appendix appeared inflamed, with wall thickening of up to 13 mm and a luminal diameter of 6 mm, consistent with appendicitis. Additional findings included multiple mesenteric lymph nodes and thickening of the ascending colon (Figure 1).

Laboratory evaluation demonstrated leukocytosis of 22,700 cells/μL, comprising 81% neutrophils, 8% lymphocytes, 10% monocytes, 1% eosinophils, and no basophils. Biochemical parameters were as follows: serum glutamic‐oxaloacetic transaminase 26 U/L, serum glutamic‐pyruvic transaminase 43 U/L, alkaline phosphatase 357 U/L, total bilirubin 0.6 mg/dL, direct bilirubin 0.2 mg/dL, serum calcium 8.4 mg/dL, and phosphorus 3.5 mg/dL. Based on clinical, radiological, and laboratory findings, a diagnosis of pyogenic liver abscess secondary to acute appendicitis was established.

**FIGURE 2:**
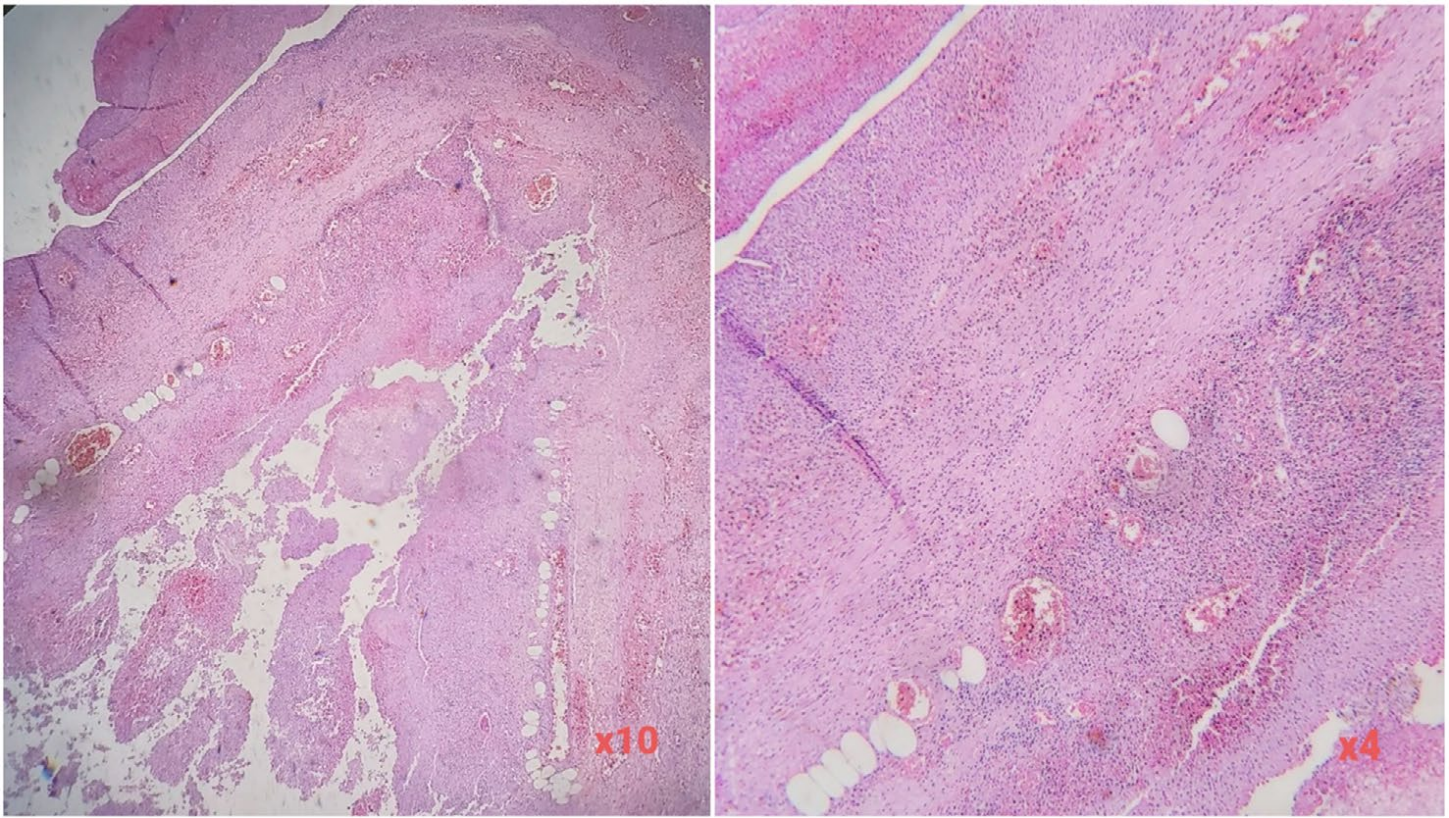
Histopathological section of the appendix demonstrating transmural inflammation with necrosis and hemorrhage (H&E, ×4 and ×10).

Surgical and radiologic consultations were obtained, and the patient subsequently underwent percutaneous drainage of the liver abscess, followed by appendectomy. Histopathological examination of the appendix revealed transmural inflammation accompanied by necrosis and hemorrhage (Figure 2). The drain was removed approximately two weeks postoperatively, once secretion had ceased and the patient's general condition had stabilized.

## 3 Differential Diagnosis

At presentation, the patient exhibited fever, altered consciousness, nausea, and vomiting, prompting consideration of several potential diagnoses. Meningitis was initially suspected and represented the primary working diagnosis; however, it was subsequently excluded following cerebrospinal fluid analysis. Pyogenic liver abscess was confirmed through CT imaging, which revealed a subcapsular hepatic collection. Other intra‐abdominal infectious etiologies, including appendicitis, cholangitis, and subphrenic abscess, were considered given the patient's gastrointestinal symptoms and imaging findings. Additionally, the possibility of hepatic malignancy or tumor was evaluated but excluded based on imaging characteristics and the clinical response to abscess drainage and antimicrobial therapy.

## 4 Conclusion and Results (Outcome and Follow‐Up)

The patient was managed with percutaneous drainage of the liver abscess and appendectomy, followed by a targeted antibiotic regimen consisting of Cefixime, Metronidazole, and Vancomycin, addressing gram‐positive, gram‐negative, and anaerobic organisms commonly implicated in intra‐abdominal infections. Microbiological culture of the abscess fluid yielded *Staphylococcus saprophyticus* , which was sensitive to amikacin and cefoxitin but resistant to vancomycin, oxacillin, erythromycin, and rifampin.

He recovered without complications and was discharged with the liver drain in situ. The drain was removed 10 days after discharge, once there was no further drainage and the patient's general condition had stabilized. At follow‐up one week later, the patient remained clinically well with complete resolution of symptoms.

Pyogenic liver abscess is a rare but serious complication of appendicitis, particularly in pediatric patients. Early imaging, prompt interventional management, and appropriate antimicrobial therapy are critical for accurate diagnosis and successful treatment. Clinicians should maintain a high index of suspicion in children presenting with atypical intra‐abdominal infection signs, ensuring timely evidence‐based management.

## 5 Discussion

Pyogenic liver abscesses occur in approximately 10–20 per 100,000 hospitalizations, or about 11 cases per million people annually. The condition predominantly affects middle‐aged individuals, peaking in the fifth and sixth decades of life due to its association with biliary disease, which remains the leading cause. In developed countries, although rare, its increasing incidence among the elderly—coupled with a globally aging population—underscores the need for greater awareness of its clinical features.

A study from Iran, similar to the present case, found that most liver abscesses in children occurred in otherwise healthy individuals. These abscesses typically presented at a younger age and were more common in boys [8]. No consistent differences in incidence have been identified based on sex, race, or geographic region. Approximately half of all cases involve a solitary abscess, most commonly in the right hepatic lobe, followed by the left and, less frequently, the caudate lobe [9].

Figure 1 demonstrates the imaging findings of this case, highlighting a subcapsular abscess in segment VI of the liver extending toward the ascending colon, which illustrates the anatomic proximity responsible for secondary infection spread from the appendix.

Management depends on abscess number and size. Smaller lesions—particularly those under 50 mm—can often be managed conservatively with antibiotics. Although bedside assessment may raise clinical suspicion, CT remains essential for evaluating abscess characteristics and identifying potential sources. In cases involving direct spread from intra‐abdominal infections, interventional or surgical treatment is frequently required alongside antimicrobial therapy. Figure 2 presents the histopathological findings of the resected appendix, confirming transmural inflammation with necrosis and hemorrhage, consistent with the underlying source of infection. Appendectomy is crucial to eliminate the ongoing source of contamination and prevent recurrence [10].

Only about 10% of patients present with the classic triad of fever, jaundice, and right upper quadrant pain. Fever is the most common symptom, often accompanied by nonspecific manifestations such as malaise, fatigue, anorexia, vomiting, weight loss, or diffuse abdominal discomfort. The condition can manifest acutely or chronically. Acute cases are often secondary to abdominal infections such as cholangitis or appendicitis, whereas chronic abscesses tend to be cryptogenic. Hematogenous abscesses typically progress rapidly (around three days), whereas those due to pylephlebitis may develop more gradually, sometimes over several weeks. Cultures from aspirated material yield positive results in 80%–90% of cases [11].

The microbial profile varies by infection source. Abscesses of biliary origin are frequently polymicrobial, while cryptogenic abscesses are often monomicrobial. *Escherichia coli* and *Klebsiella pneumoniae* are the predominant pathogens. Community‐acquired *K. pneumoniae* liver abscesses, typically monomicrobial, have become a major public health concern in parts of Asia, responsible for up to 80% of cases in Taiwan and South Korea, with increasing reports globally. Despite relatively low mortality, metastatic complications—including meningitis and endophthalmitis—occur in up to 16% of cases. The diagnosis should be considered in any patient presenting with fever, leukocytosis, and hepatic lesions on imaging [12].

Due to nonspecific early symptoms, misdiagnosis is frequent, with differential diagnoses including cholangitis, pneumonia, hepatic malignancy, or other intra‐abdominal infections. Leukocytosis is present in 68%–88% of patients, typically ranging from 15,000 to 17,000 cells/μL. Elevated alkaline phosphatase is the most frequent laboratory abnormality, though observed in only about 2.3% of cases. Normal liver enzyme and bilirubin levels do not exclude the diagnosis. Albumin and prothrombin time values are usually within normal limits [13].

Chest radiography is abnormal in approximately half of all cases but has limited diagnostic specificity. A high index of suspicion is essential for early recognition. Hemoglobin levels below 10 g/dL and blood urea nitrogen (BUN) levels above 28 mg/dL have been identified as independent mortality predictors. Imaging remains critical for diagnosis. Ultrasound and CT are the main diagnostic modalities; ultrasound is particularly useful in patients unable to receive contrast or with suspected biliary disease. Ultrasound sensitivity ranges from 70% to 90%, while CT offers up to 95% sensitivity and is preferred for complex drainage planning. Magnetic resonance imaging (MRI) is rarely necessary but may assist in differentiating abscesses from noninfectious hepatic lesions such as neoplasms. Fine‐needle aspiration (FNA) remains the diagnostic gold standard. Blood cultures are positive in approximately 50% of cases, and multiple aerobic and anaerobic cultures should be obtained [14].

A definitive diagnosis is established through aspiration of purulent material, typically performed under imaging guidance. If no purulent material is retrieved, alternative diagnoses—such as hepatic cysts, tumors, or sterile abscesses—should be considered. Aspirates should be sent for Gram staining, which can provide early diagnostic clues, particularly in amebic infections. Management generally requires both abscess drainage and appropriate antibiotic therapy. Advances in imaging have made image‐guided percutaneous drainage the preferred first‐line intervention. When open drainage is not performed, close clinical monitoring is essential to ensure improvement and prevent recurrence [15].

If percutaneous drainage fails, surgical intervention or management of the underlying intra‐abdominal source should be pursued. Surgery may also be indicated for multiloculated, large, or multiple abscesses. Once a pyogenic liver abscess is suspected, empirical broad‐spectrum antibiotics should be initiated promptly. Although blood cultures should be obtained before starting antibiotics, treatment should not be delayed for aspiration. Biliary‐source abscesses usually contain enterococci and gram‐negative bacilli; those originating from colonic or pelvic infections involve gram‐negative and anaerobic organisms. Hematogenous infections often require coverage for *Staphylococcus aureus* . Standard therapy includes two to three weeks of intravenous antibiotics, followed by four to six weeks of oral therapy [16]. In selected cases, shorter regimens may be effective.

Beyond pyogenic abscesses, hepatic infections may also arise from amebic or fungal pathogens, each exhibiting both overlapping and distinct clinical and epidemiological profiles. All three types typically involve the liver parenchyma and manifest with nonspecific systemic symptoms such as fever and abdominal pain [17, 18, 19]. Imaging modalities—including ultrasound and CT scanning—remain essential diagnostic tools across all etiologies [20]. Similarly, image‐guided percutaneous drainage, by either aspiration or catheter placement, is a standard therapeutic approach for bacterial and amebic abscesses, particularly for larger or symptomatic lesions [14, 18, 20].

Despite these similarities, key differences exist. Pyogenic abscesses are most commonly caused by *Klebsiella pneumoniae* and are frequently associated with biliary pathology [21, 22]. Amebic abscesses, in contrast, result from infection with *Entamoeba histolytica* and remain endemic in regions with poor sanitation and fecal–oral transmission [23, 24]. Fungal abscesses, though rare—accounting for less than 2% of hepatic abscesses—typically arise from Candida or Aspergillus species and occur predominantly in immunocompromised hosts, such as those with hematologic malignancies or advanced liver disease [21, 24, 25, 26, 27]. Clinically, bacterial and amebic abscesses often present with high‐grade fever and right upper quadrant pain, whereas fungal abscesses may display an indolent course with nonspecific features, such as prolonged fever of unknown origin in immunocompromised patients [21, 25].

Although most liver abscesses respond well to timely drainage and antibiotic therapy, delayed or inadequate management can lead to serious complications. Among these, suppurative portal vein thrombosis (pylephlebitis) is a particularly severe consequence, resulting from septic thrombosis of the portal venous system secondary to intra‐abdominal infections such as appendicitis or diverticulitis [28, 29]. Additional complications include pleural effusion, peritonitis following abscess rupture, and systemic septic shock [30, 31]. These sequelae substantially increase morbidity and hospitalization duration, often arising from diagnostic delays or incomplete source control [32]. Therefore, prompt recognition and aggressive management are essential to prevent progression. Clinicians should maintain vigilance for vascular or systemic dissemination, particularly in patients showing poor clinical response despite appropriate therapy [33, 34].

Follow‐up should be guided by both clinical and radiologic response to determine the appropriate duration of antibiotic therapy and the need for re‐aspiration. Although most abscess cavities resolve with adequate treatment, some may persist. Recurrence of symptoms such as fever or abdominal pain should prompt repeat imaging and re‐aspiration if indicated [15].

**FIGURE 3:**
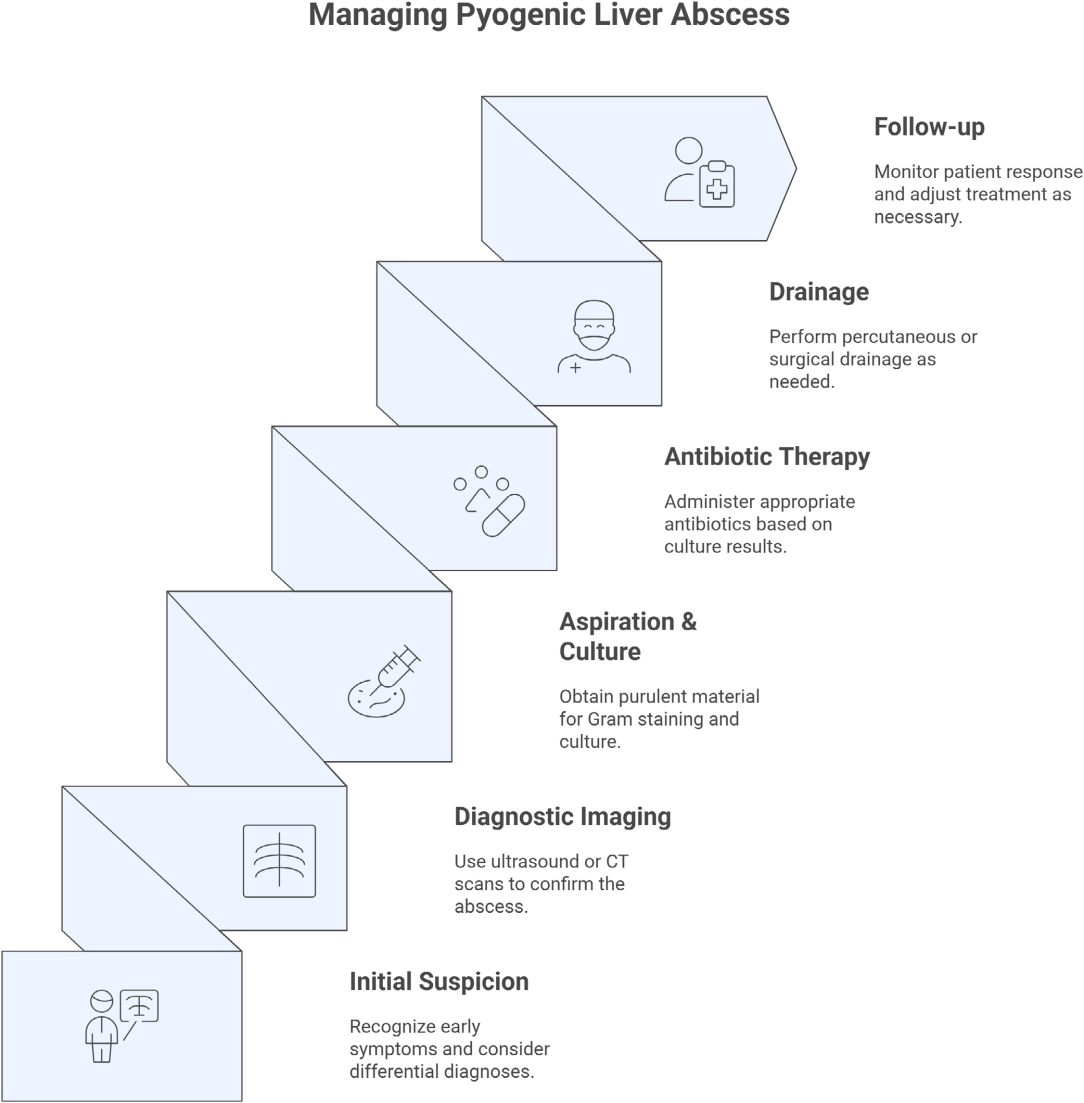
Steps in the management of pyogenic liver abscess.

Figure 3 illustrates the stepwise management approach for pyogenic liver abscess, summarizing diagnostic evaluation, initial stabilization, image‐guided drainage, targeted antimicrobial therapy, and criteria for surgical intervention. This algorithm emphasizes a multidisciplinary, evidence‐based approach, facilitating early recognition, effective treatment, and prevention of recurrence, thereby enhancing the educational value of this case report.

## Author Contributions

**Mohammad AshrafAzimi:** project administration, resources, writing – original draft. **Abbas Abdollahi:** supervision. **Mohammad karimi:** investigation, resources. **Homa Jajarmi:** investigation, project administration, resources. **Ali Mehri:** project administration, visualization, writing – original draft, writing – review and editing.

## Funding

The authors received no financial support for this study.

## Ethics Statement

Surgery and all other procedures were done with the agreement of the 1975 Helsinki Declaration, and informed consent was obtained from the patient.

## Consent

Written informed consent was obtained from the patient to publish this report in accordance with the journal's patient consent policy.

## Conflicts of Interest

The authors declare no conflicts of interest.

## Data Availability

Data sharing not applicable to is this article as no datasets were generated or analysed during the current study.
